# Elongational Flow Field Processed Ultrahigh Molecular Weight Polyethylene/Polypropylene Blends with Distinct Interlayer Phase for Enhanced Tribological Properties

**DOI:** 10.3390/polym13121933

**Published:** 2021-06-10

**Authors:** Xiaochuan Chen, Xiaotong Wang, Changlin Cao, Zhongke Yuan, Dingshan Yu, Fei Li, Xudong Chen

**Affiliations:** 1 Key Laboratory for Polymeric Composite and Functional Materials of Ministry of Education, School of Chemistry, Sun Yat-Sen University, Guangzhou 510275, China; chenxch29@mail2.sysu.edu.cn (X.C.); wangxt58@mail2.sysu.edu.cn (X.W.); yuanzhk3@mail.sysu.edu.cn (Z.Y.); 2 Key Laboratory of High Performance Polymerbased Composites of Guangdong Province, School of Chemistry, Sun Yat-Sen University, Guangzhou 510275, China; 3 College of Environmental Science and Engineering, Fujian Key Laboratory of Pollution Control & Resource Reuse, Fujian Normal University, Fuzhou 350007, China; caochlin3@fjnu.edu.cn; 4 Engineering Research Center of Polymer Green Recycling of Ministry of Education, Fujian Normal University, Fuzhou 350007, China

**Keywords:** eccentric rotor extruder, UHMWPE, wear, mechanical properties

## Abstract

Herein, we produced a series of ultrahigh molecular weight polyethylene/polypropylene (UHMWPE/PP) blends by elongational-flow-field dominated eccentric rotor extruder (ERE) and shear-flow-field dominated twin screw extruder (TSE) respectively and presented a detailed comparative study on microstructures and tribological properties of UHMWPE/PP by different processing modes. Compared with the shear flow field in TSE, the elongational flow field in ERE facilitates the dispersion of PP in the UHMWPE matrix and promotes the interdiffusion of UHMWPE and PP molecular chains. For the first time, we discovered the presence of the interlayer phase in blends with different processing modes by using Raman mapping inspection. The elongational flow field introduces strong interaction to enable excellent compatibility of UHMWPE and PP and induces more pronounced interlayer phase with respect to the shear flow field, eventually endowing UHMWPE/PP with improved wear resistance. The optimized UHMWPE/PP (85/15) blend processed by ERE displayed higher tensile strength (25.3 MPa), higher elongation at break (341.77%) and lower wear loss of ERE-85/15 (1.5 mg) compared to the blend created by TSE. By systematically investigating the microstructures and mechanical properties of blends, we found that with increased content of PP, the wear mechanism of blends varies from abrasive wear, fatigue wear, to adhesion wear as the dominant mechanism for two processing modes.

## 1. Introduction

Ultrahigh molecular weight polyethylene (UHMWPE) has multiple advantages including good self-lubricating ability, low friction coefficient, high impact strength, fatigue resistance, and biological inertness, which demonstrates its potential use as a wear-resistant material in industrial bearings, protective layer, and artificial joints [1]. However, owing to the relatively high average of UHMWPE, irregular inter-chain entanglement results in high regional chain density and low mass flow rate (MFR), making UHMWPE difficult to be processed by common injection molding or extrusion processes. In addition, the low surface hardness, low modulus of elasticity, and bending strength, and poor abrasion resistance of UHMWPE greatly limit its application [2]. It is therefore of great significance to develop industrially viable UHMWPE-based wear resistant materials through the regulation of polymer composition and the optimization of processing technology [3].

Recently, developing UHMWPE-based blends, i.e., introducing low density polyethylene (LDPE), Poly (lactic acid) (PLLA), poly (ethylene oxide) (PEO), etc., into the UHMWPE matrix, is a viable option to improve the processability [4,5,6,7]. However, excessive amounts of additives will lead to an increased mobility of the UHMWPE based blends, while its mechanical properties may be adversely affected [8,9]. Due to the superiority of polypropylene (PP) with high MFR value, a mixture of a certain amount of PP with UHMWPE also can effectively improve the processing properties of UHMWPE-based blends [10]. Unlike high density polyethylene (HDPE), which penetrates the UHMWPE particles, PP acts as a lubricant which is distributed between the primary and secondary particles of UHMWPE, enhancing the processing properties, but with limited improvements in wear resistance and mechanical properties [11]. Moreover, changing the strain type of the forming process can also be used to improve the target performance of the blends [12,13]. The flow field of polymer materials in processing is divided into a shear flow field where the velocity gradient direction is in line with the flow direction and the elongational flow field where the velocity gradient direction is perpendicular to the flow direction, corresponding to the twin-screw extruder (TSE) and eccentric rotor extruder (ERE), respectively. The different flow fields greatly influence the compatibility of the two phases in the blends, giving rise to differences in all aspects of performance [14,15,16,17]. Therefore, the relationship between the structure and the wear resistant properties of the blends in ERE and TSE needed to be systematically investigated.

As is well known, one marked difference between polymers and metallic wear-resistant materials is that polymers tend to have a higher viscoelasticity and that wear volume loss in polymers is a complex behavior that is influenced by many factors such as friction type, strength, resistance, temperature, and geometry of friction nodes [18,19]. The common wear mechanisms in polymers are adhesive wear, abrasive wear, and fatigue wear, in which fatigue wear differs significantly from adhesive or abrasive wear and does not cause significant damage to the surface until a critical number of cycles is reached [20]. The wear resistant properties of polymers are generally determined to some extent by their chemical structures, while the processing technology exerts a great influence on the chemical structure of polymers [21,22]. In order to ensure that the physical and chemical properties of the products meet the required conditions, it is necessary to provide a lucid understanding on morphological structure of the products.

In this contribution, we prepared a series of UHMWPE/PP blends with different composition by two processing methods TSE and ERE respectively and provided a comparative study of structures and mechanical and wear resistance properties of blends processed by elongational flow field and shear flow field. With the same PP content, the elongation strength of ERE-85/15 (25.3 MPa) was higher than that of TSE-85/15 (22.7 MPa), while the elongation at break (341.77%) was four times higher. In the sliding wear test, the wear volume loss of ERE-85/15 (1.5 mg) was less than that of TSE-85/15 (3.0 mg), which indicates that the ERE-processed blend has superior mechanical and wear resistant properties. We also elucidated the enhancement mechanism by Raman spectroscopy and SEM, showing that the elongational flow field facilitates the dispersion of PP phase in the UHMWPE matrix and promotes the interdiffusion of UHMWPE and PP molecular chains, resulting in the formation of a wide interlayer phase (1~2 μm). This ‘soft link’ interlayer phase means that the chains of UHMWPE and PP are partially entangled under elongational flow field and effectively strengthens the interface between the two phases, endowing the UHMWPE/PP blend better wear resistance.

## 2. Materials and Methods

### 2.1. Materials

UHMWPE GUR4120, viscosity-averaged relative molecular mass 4.7 × 10^6^ g/mol, Tekona GmbH, Germany; PP: Z30s, viscosity-averaged relative molecular mass 1.5 × 10^5^ g/mol, Maoming Petrochemical Co. (Guangdong, China).

### 2.2. Sample Preparation

UHMWPE/PP blends were processed by ERE (ERE-30-CV-A, Guangdong Xinglian Precision Machinery Co., Ltd., Guangdong, China) and TSE (MEDI-22/40, Guangzhou Putong Experimental Analytical Instruments Co., Ltd., Guangdong, China).

Briefly, the UHMWPE/PP blend was processed by using ERE with the UHMWPE/PP mass ratios of 100/0, 95/5, 85/15, 75/25, 65/35, and 50/50 at a speed of 25 rpm and various processing temperatures of 200 °C, respectively. The extrusion die was an 8 mm round bar mill; the samples were extruded from the ERE and immediately placed in a stainless-steel die and pressed into sheets at 17 MPa.

The UHMWPE/PP blend was also produced by TSE. The polymers with similar mass ratio as that processed by the ERE were put into a TSE for melt blending with a screw diameter of 21.7 mm, a speed of 220 rpm and a melt temperature of 200 °C. The samples were extruded by the twin-screw and immediately placed in a stainless-steel die and pressed into sheets at a pressure of 17 MPa. Pure UHMWPE was also processed into CM-UPE consists of pure UHMWPE, which is processed directly by compression molding (CM) using a flat plate vulcanizer (Flat Plate Vulcanizer: ZG-80T, Dongguan Zhenggong Mechanical & Electrical Equipment Technology Co., Dongguan, China).

### 2.3. Sample Characterization

The morphology and structure were examined by scanning electron microscopy (SEM: HITACHI-Regulus 8100, Tokyo, Japan). Confocal Raman spectroscopy (DXR2xi, Thermo Scientific) was employed to investigate microstructures and interface phase morphology with a 532 nm argon ion laser.

The crystallinity of blends was acquired by differential scanning calorimetry (DSC: Q20, TA, USA). The heating and cooling rate of the whole test process was set at 10 °C/min. Dynamic thermomechanical analysis (DMA: Q800, TA Instruments, New Castle, DE, USA) was used to analyze the thermo-mechanical properties of the samples with a temperature range of 30–180 °C, a temperature rise rate of 3 °C/min, an amplitude of 5 µm and a test frequency of 1 Hz. The samples were tested for mechanical properties according to GB/T 1040.2-2006 standard, with the elongational speed set at 50 mm/min and at room temperature.

The tensile strength and elongation at break were performed using microcomputer-controlled electronic universal testing machine (CMT4104, Shenzhen New Sansi Materials Testing Co., Shenzhen, China). The M-200 plastic sliding friction and wear tester was used to determine the sliding friction and wear properties of the material according to the standard GB/T 3960-2006. The sample size was 30 mm × 7 mm × 6 mm, and the test was carried out under dry friction conditions on 45# steel with a speed of 200 r/min. The temperature of the metal grinding wheel surface was monitored at 30 min intervals using an infrared thermometer.

## 3. Results and Discussion

To achieve the imaging of fracture surface, the sample was completely submerged in liquid nitrogen for a certain period and then, after complete freezing, an isotropic stress was applied at both ends of the sample, resulting in a fracture in the central stress concentration area of the sample.

The crystallinity of blends was acquired by DSC, and the crystallinity of UHMWPE was calculated according to Equation (1):

(1) $$X_{c}=\frac{∆H_{m}}{∆H_{m_{0}}\times \emptyset_{m}}\times 100\%$$

where ∅_m_ as the mass fraction of UHMWPE; $\Delta H_{m}$ as the enthalpy of melting of UHMWPE; $\Delta H_{m_{0}}$ represents the enthalpy of melting when $X_{c}$ is 100%, and its value is 290 J/g [23]. Based on the Raman spectra of the microstructure of the samples, the crystallinity (*X_c_*) is calculated according to Equation (2) [24]:

(2) $$X_{c}=\frac{I_{1416}}{0.46\times I_{\left(1295+1305\right)}}$$

The phase morphology is indicative of blends was derived from Equation (3) [25]:

(3) $$Phase\;morphology=\frac{I_{804}}{I_{1295}}$$

where *I* denotes the peak area corresponding to the position of the Raman peak. *I*_804_ and *I*_1295_ are the indications of PP and UHMWPE, respectively.

**Figure 1:**
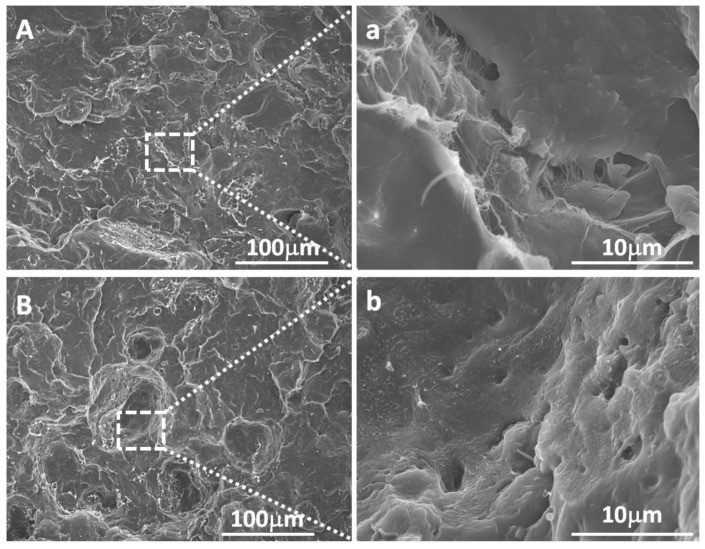
SEM images of fracture of UHMWPE/PP 85/15: by TSE (**A**,**a**). and by ERE (**B**,**b**). (**a**,**b**) represent the corresponding enlarged area in (**A**,**B**).

UHMWPE/PP blends with various mass ratios of 100/0, 95/5, 85/15, 75/25, 65/35, and 50/50 were extruded into sheets using the ERE and TSE, respectively. Figure 1 and Figure S1 (see Supplementary Materials) shows typical SEM images of the fractural surface of the UHMWPE/PP blends processed by ERE and TSE. As seen from Figure 1, the fractured surface of UHMWPE/PP by TSE exhibits a high density of cavities. A higher magnification of SEM imaging reveals that the cavity wall is smooth, and the two-phase interface is clearly visible. This could be caused by the poor compatibility of UHMWPE and PP and the weak interfacial bonding strength under the shear flow field dominated in TSE (Figure 1A,a). In contrast, for UHMWPE/PP extruded by ERE, the cavity number on the fractured surface is markedly reduced and the cavity wall becomes rougher with fibril-like structure exposed, while the two-phase interface is more blurred (Figure 1B,b). This indicates that PP is more uniformly dispersed in the UHMWPE matrix with the stronger interfacial bonding strength induced by the elongational flow field.

**Figure 2:**
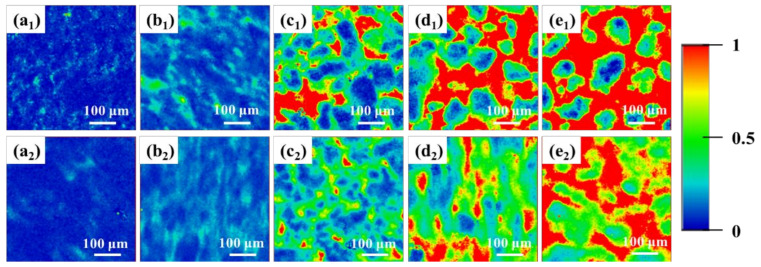
Raman mapping of ratio of the peak intensities (I_804_/I_1295_) for UHMWPE/PP: (**a**) 95/5, (**b**) 85/15, (**c**) 75/25, (**d**) 65/35, (**e**) 50/50; **1** and **2** stand for the UHMWPE/PP prepared by shear flow and elongational flow, respectively.

As observed in Figure S2 (see Supplementary Materials), the Raman spectrum of pure UHMWPE shows a characteristic peak at 1295 cm^−1^ assigned to the twisted vibration of CH_2_, while pure PP reveals a characteristic peak at 804 cm^−1^, attributable to the wobble vibration of CH_2_ [26]. The distribution of UHMWPE and PP in the blends can be obtained by fitting the Raman data according to Equations (1)–(3) mentioned above. As shown in Figure 2, the red part in the Raman mapping means a larger I_PP_/I_UHMWPE_ ratio, indicating that the red region is dominated by the PP phase. The blue part denotes a smaller I_PP_/I_UHMWPE_ ratio, implying the dominant presence of the UHMWPE phase. The green part suggests the coexistence of equivalent UHMWPE and PP phases. Under either elongational or shear flow fields, as the PP content is less than 25%, UHMWPE act as the continuous phase while PP is dispersed in the UHMWPE matrix to form an island structure (Figure 2a_1_,a_2_,b_1_,b_2_). As the PP content approaches 25%, the area of the green region increases and a bi-continuous structure form (Figure 2c_1_,c_2_). When the PP content is above 25%, the area of the red area gradually increases while the area of the blue area decreases (Figure 2d_1_,d_2_,e_1_,e_2_), indicating a reverse blend system with PP as the continuous phase and UHMWPE as the dispersed phase. Having established that ERE dominated by elongational stress can induce a uniformly dispersed phase morphology of PP. In contrast, PP under the action of shear flow field is more likely to agglomerate in the UHMWPE matrix and the UHMWPE/PP blend is prone to phase separation.

DSC curves of UHMWP/PP blends obtained by different processing methods are shown in Figure S3a,b (see Supplementary Materials). The UHMWPE/PP blends extruded under the elongational flow field exhibit two melt peaks at near ~135 °C (UHMWPE) and 164 °C (PP), respectively, while the melt peak of PP becomes apparent with the increase of the PP content. Moreover, the melt enthalpy and crystallinity of UHMWPE decrease with the addition of PP, suggesting that UHMWPE/PP is an incompatible system, and the two phases compete during the crystallization process. Interestingly, the melting point of PP in the UHMWPE/PP blend by ERE (~164 °C) is higher than that of PP in the blend prepared by TSE (~155 °C) [27,28]. The cooling crystallization curves demonstrate that only one crystallization peak appears in the UHMWPE/PP blend obtained under the elongational flow field, while the blend sample from the shear flow field feature a double peak appears when the PP content is >25% (Figure S3c,d, see Supplementary Materials). Moreover, the crystallinity analysis derived from the DSC results confirms that there is no significant difference in the crystallinity variation of the blend samples produced by TSE and ERE (Table S1, see Supplementary Materials).

The tensile strength and elongation at break of the UHMWPE/PP blends processed by ERE and TSE are shown in Figure S4 (see Supplementary Materials). Clearly, in terms of the tensile strength and elongation at break, the UHMWPE/PP blends by ERE exhibit similar variation trend with the increased PP content compared to those by TSE, while the ERE-processed blends display much higher tensile strength and elongation at break (i.e., 25.31 MPa and 341.77% at 15% PP content) with respect to the TSE-processed counterpart (22.71 MPa and 85.13%). This result highlights the distinct advantage of the elongational flow field for superior mechanical performance in the UHMWPE/PP blends.

**Figure 3:**
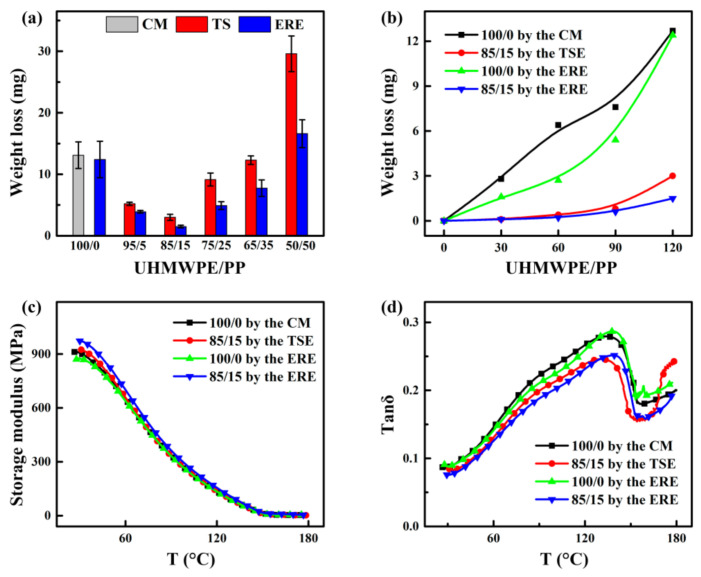
(**a**) Weight loss of UHMWPE and UHMWPE/PP prepared under different processing methods (2 h); (**b**) weight loss-time curve; DMA curves of UHMWPE and UHMWPE/PP by different processing methods: (**c**) storage modulus; (**d**) loss factor (Tanδ).

Figure 3a presents the wear loss of the UHMWPE and UHMWPE/PP blends with different processing methods after 2 h of wear test. For the UHMWPE blends by either ERE or TSE, the optimal PP content to yield the best tribological property is 15% (the UHMWPE/PP ratio: 85/15), while the optimized ERE-processed blend produces lower wear loss (1.5 mg) relative to the TSE-processed blend (3.0 mg), highlighting the superiority of the elongational flow field—dominated processing. Interestingly, such weight loss values from the blends are significantly lower than those of pure UHMWPE samples produced by CM and ERE (13.1 and 12.4 mg) under identical wear test conditions, verifying the crucial role of the introduced PP within the UHMWPE matrix toward the tribological property. On the other hand, as indicated by the weight loss time profiles in Figure 3b, the wear degree for the UHMWPE blends produced by either ERE or TSE shows very small variations with increased time throughout the whole friction period in stark contrast to pure UHMWPE showing marked increase with increased friction time. This result implies that the blends are less sensitive to the friction time compared to pure UHMWPE. An analysis of the tribological performance based on UHMWPE/PP reveals that the wear resistance of UHMWPE increases significantly when the PP content is 15%. The relationship curves of friction coefficient—the time for pure UHMWPE and UHMWPE/PP (85/15) indicates that the variation trend for samples with different processing methods is basically the same (Figure S5, see Supplementary Materials). In the initial stage (0–2400 s), the surface friction coefficient increases sharply, then, it shows a slow increase, and after 7200 S, the coefficient of friction of ERE-85/15 (0.35) and TSE-85/15 (0.36) containing pp is significantly smaller than that of CM-UPE (0.37) and ERE-UPE (0.38) without PP. This is most likely since the lateral methyl group contained in the structural formula of PP makes the hardness and modulus of PP larger than that of UHMWPE, which improves the ability of UHMWPE to resist plastic deformation and reduces the contact area between the friction, which in turn reduces the friction.

The investigation of the surface morphology of the sample after rubbing can intuitively assess the wear degree of the sample and the corresponding wear mechanism [19,29]. As shown in Figure S6 (see Supplementary Materials), the wear track of the blends with low PP content (<5%) shows many furrows, known as abrasive wear, while the surface morphology of the blend with medium PP content (5–25%) features small, shallow depressions or plastic deformations, which are caused by contact stress. The wear surface of the samples with high PP content (25–50%) exhibits tears, which is resulted from the adhesion between the sample surface and the counter-abrasive surface; the wear mechanisms corresponding to these three morphologies are, respectively, abrasive wear, fatigue wear and adhesive wear. the UHMWPE/PP blend prepared by TSE and ERE has a similar friction morphology but is clearly different from pure UHMWPE. The wear tracks show significantly less furrows, indicating that the addition of PP improves the wear resistance of UHMWPE to a certain extent. In addition, it is noted that under the action of the shear flow field, the wear surface of the UHMWPE/PP blend also exhibits more fatigue cracks when the PP content was >25%, where the wear behavior is in the form of adhesive wear and fatigue wear. In contrast, the cracks in the UHMWPE/PP blend prepared by ERE only appears when the PP content was >35%. Because the interfacial bonding strength of the UHMWPE/PP blends prepared by TSE is weaker than of the blends prepared by ESE, the material is prone to tear under wear process, thus triggering cracks.

Dynamic thermomechanical analysis (DMA) result in Figure 3c reveals that, with increased temperature, the storage modulus for all the samples including pure UHMWPE and the UHMWPE/PP blends by different processing exhibit an initial dramatic decrease, followed by the appearance of a stable plateau at high temperature. Of note, at lower temperature the ERE-processed UHMWPE/PP (85/15) blend presents higher storage modulus with respect to other counterparts, indicating better resistance to plastic deformation and yield stress. The corresponding loss factor (Tanδ)—temperature curve in Figure 3d indicates that the UHMWPE/PP blend exhibits lower Tanδ than those of pure UHMWPE.

Based on the analysis of the dynamic thermomechanical properties of UHMWPE/PP, the surface temperature of the material during the wear process was examined using an infrared thermometer, and the results are shown in Figure S7 and Table S2 (see Supplementary Materials). CM-100/0 and ERE-100/0 have a similar temperature evolution, both reaching surface temperatures of around 140 °C after 120 min of testing, which exceeded the melting point temperature of UHMWPE (~135 °C), and was significantly higher than TSE-85/15 (127 °C) and ERE-85/15 (124 °C), suggesting that the PP content has ability to optimize thermal conductivity and decrease coefficient of friction, which facilitates to reduce the friction temperature of the UHMWPE/PP surface and mitigate the degree of oxidative degradation. 

**Figure 4:**
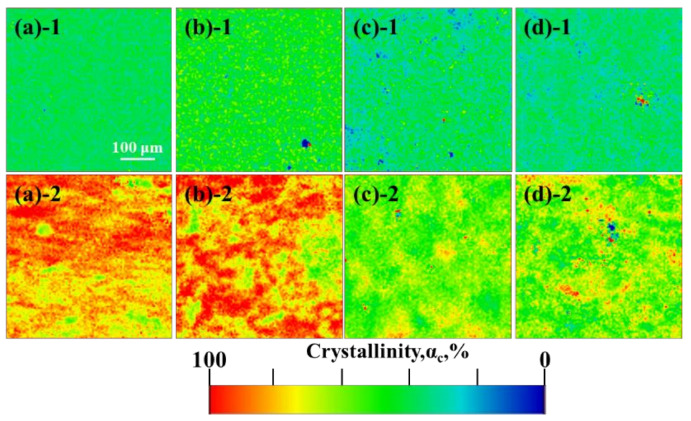
Surface crystallinity distribution of Raman mapping of UHMWPE and UHMWPE/PP under different processing methods: (**a**) CM-UPE; (**b**) ERE-UPE; (**c**) ERE-85/15; (**d**) TSE-85/15; “**1**” and “**2**” stand for before friction and after friction, respectively.

Raman mapping plots of the surface crystallinity distribution of the samples before and after sliding friction testing are shown in Figure 4. The crystallinity of CM-UPE, ERE-UPE, ERE-85/15, and TSE-85/15 are basically distributed around 50% before wear. After sliding wear testing, the mapping distribution of all samples partly shifted towards red, i.e., the crystallinity increased, which result from orientation-induced crystallization of the surface. For the ERE-85/15 and TSE-85/15, the increase in crystallinity was significantly smaller than that of the CM-UPE and ERE-UPE, demonstrating the high friction temperature promotes the rearrangement of polymer chain, while ERE-85/15, and TSE-85/15 remain stable during friction due to its low coefficient of friction and good thermal conductivity. 

**Figure 5:**
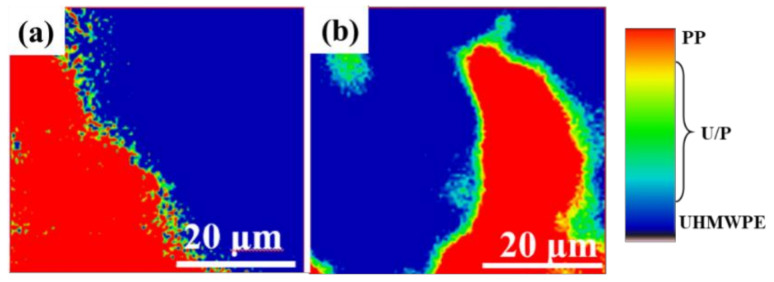
Raman mapping of ratio of the peak intensities (I_804_/I_1295_) for UHMWPE/PP under different processing methods: (**a**) by shear flow; (**b**) by elongational flow.

**Figure 6:**
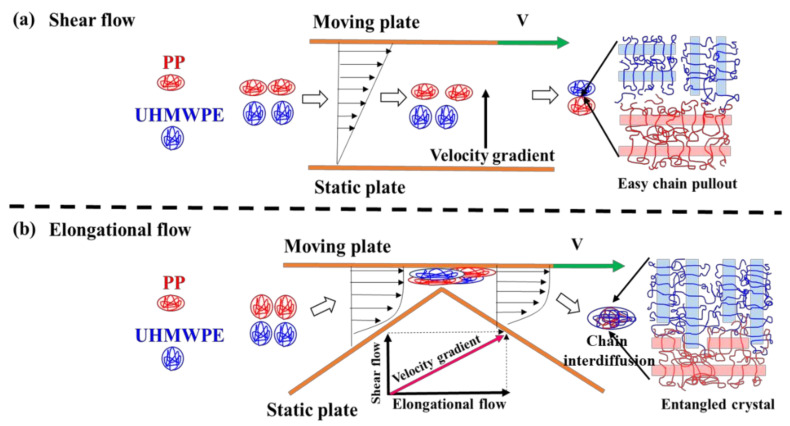
Proposed schematic representation of UHMWPE/PP interfaces by different processing methods: (**a**) by shear flow; (**b**) by elongational flow.

The above results confirm that the PP content in UHMWPE matrix plays a crucial role in enforcing the mechanical properties and wear resistant performance, while the differences in frictional properties triggered by TSE and ERE need to be explored in more depth. The interfacial microstructure of the UHMWP/PP blend was further characterized by the high-resolution Raman mapping (Figure 5). Clearly, the UHMWP/PP blend extruded by ERE displays a thicker interphase (1–2 μm) compared to the sample produced by TSE, which attributes to better compatibility of blends processed by ERE. We propose the reason for strong interactions based on the principles underlying the process; as shown in Figure 6a, under the shear flow field in TSE, the velocity gradient is perpendicular to the direction of the flow field, which tends to form a flow with weak interaction between the layers. Thus, it is difficult for the molecular chains of UHMWPE and PP to diffuse with each other. When subjected to the elongational flow field in ERE, the introduction of a compressive stress perpendicular to the flow field tends to result in strong interaction between UHMWPE and PP and thus the molecular chains easily diffuse and entangle each other (Figure 6b). Owing to the long relaxation time of the molecular chains, phase separation of blends is difficult to form during the cooling period and cause entanglement of the interfacial molecular chains, increasing the interfacial bonding strength and greatly improving the target performance of the material processed by ERE.

## 4. Conclusions

UHMWPE/PP with different component ratios developed by TSE and ERE processing techniques were systematically investigated for wear-resistant properties and we found that PP was able to reduce the loss factor (Tan δ) and friction coefficient of the UHMWPE matrix, contributing to the maintenance of good mechanical properties of the composite during friction, resulting in less oxidative degradation of the friction surface and less increase in crystallinity of the friction surface. Importantly, the elongational flow field clearly contributed to the ability of PP reinforcement to disperse in the UHMWPE matrix, facilitating the interdiffusion of UHMWPE and PP molecular chains, and the formation of a wider intercalation phase (approximately 2 μm) was demonstrated for the first time by Raman mapping techniques. This interlayer phase effectively strengthens the interface between UHMWPE and PP and dispersed the stress from the surface layer to a wider area during wear process, giving the UHMWPE/PP blend superior wear resistance compared to the interface created by the TSE process. The elongational strength of ERE-85/15 (25.3 MPa) was higher than that of TSE-85/15 (22.7 MPa) with the same PP content, while the elongation at break (341.77%) was four times higher and the wear volume loss of ERE-85/15 (1.5 mg) is less than of TSE-85/15 (3.0 mg) in the sliding wear test. This new hybrid composite material developed using ERE processing has excellent tribological properties that can be expanded into many new applications such as advanced structural materials, protective coatings for micromechanical systems and components that are resistant to contact damage.

## Data Availability

The data presented in this study are available upon request from the corresponding authors.

## Supplementary Materials

The following are available online at https://www.mdpi.com/article/10.3390/polym13121933/s1, Figures S1–S7, Tables S1–S2. Figure S1: SEM images of cryofractured surfaces of UHMWPE/PP Figure S2: Raman spectra of UHMWPE and PP, Figure S3: DSC curve of UHMWPE/PP under different processing methods Figure S4: Mechanical properties of UHMWPE/PP under different processing methods Figure S5: Curve of friction coefficient as a function of friction time of UHMWPE and UHMWPE/PP under different processing methods, Figure S6: SEM images of the end of the friction test of UHMWPE and UHMWPE/PP. Figure S7: Friction temperature of UHMWPE and UHMWPE/PP under different processing, Table S1: DSC related data of UHMWPE by different processing methods, Table S2: Surface frictional temperature by different friction time (°C).
