# Supplementary material for: Elongational Flow Field Processed Ultrahigh Molecular Weight Polyethylene/Polypropylene Blends with Distinct Interlayer Phase for Enhanced Tribological Properties
Source: Polymers (Basel). 2021 Jun 10;13(12):1933. doi: 10.3390/polym13121933 (PMC8230468; doi:10.3390/polym13121933)
Supplement: Supplementary file 1 [file polymers-13-01933-s001.zip › polymers-1249064-supplementary.pdf]

Elongational Flow Field Processed Ultrahigh Molecular Weight Polyethylene/Polypropylene Blends with Distinct Interlayer Phase for Enhanced Tribological Properties

Xiaochuan Chen [1,2,†], Xiaotong Wang [1,2,†], Changlin Cao [3,4], Zhongke Yuan [1,2], Dingshan Yu [1,2,*], Fei Li [3,4,*] and Xudong Chen [1,2,*]

1 Key Laboratory for Polymeric Composite and Functional Materials of Ministry of Education, School of Chemistry, Sun Yat-Sen University, Guangzhou 510275, China; chenxch29@mail2.sysu.edu.cn (X.C.); wangxt58@mail2.sysu.edu.cn (X.W.); yuanzhk3@mail.sysu.edu.cn (Z.Y.)
2 Key Laboratory of High Performance Polymerbased Composites of Guangdong Province, School of Chemistry, Sun Yat-Sen University, Guangzhou 510275, China
3 College of Environmental Science and Engineering, Fujian Key Laboratory of Pollution Control & Resource Reuse, Fujian Normal University, Fuzhou 350007, China; caochlin3@fjnu.edu.cn
4 Engineering Research Center of Polymer Green Recycling of Ministry of Education, Fujian Normal University, Fuzhou 350007, China
* Correspondence: yudings@mail.sysu.edu.cn (D.Y.); lifei1@cgnpc.com.cn (F.L.); cescxd@mail.sysu.edu.cn (X.C.)
† These authors contributed equally to this work.

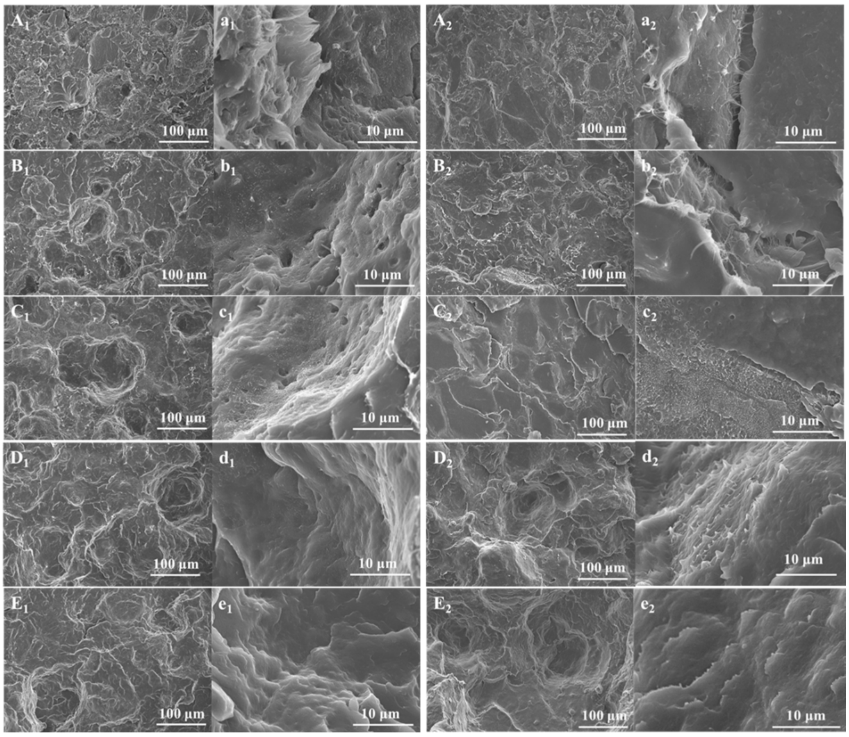

Figure S1. SEM images of cryofractured surfaces of UHMWPE/PP: (A, a) 95/5, (B, b) 85/15, (C, c) 75/25, (D, d) 65/35, (E, e) 50/50; "1" and "2" stand for the UHMWPE/PP prepared by shear flow and elongational flow respectively, a-e represent the corresponding enlarged area in A-E.

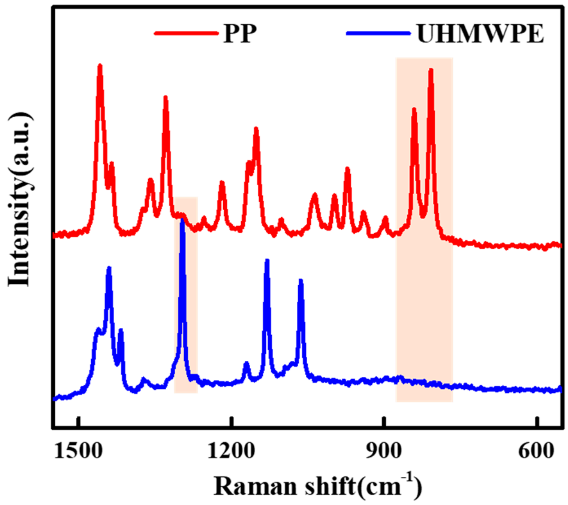

Figure S2. Raman spectra of UHMWPE and PP.

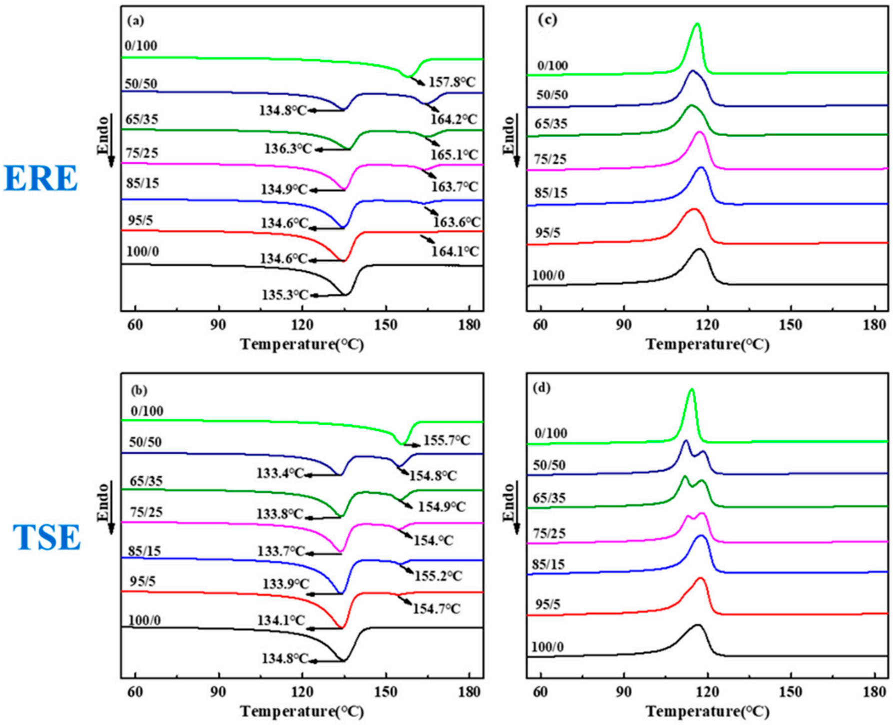

Figure S3. DSC curve of UHMWPE/PP under different processing methods: (a-b) secondary heating curve; (c-d) cooling curve.

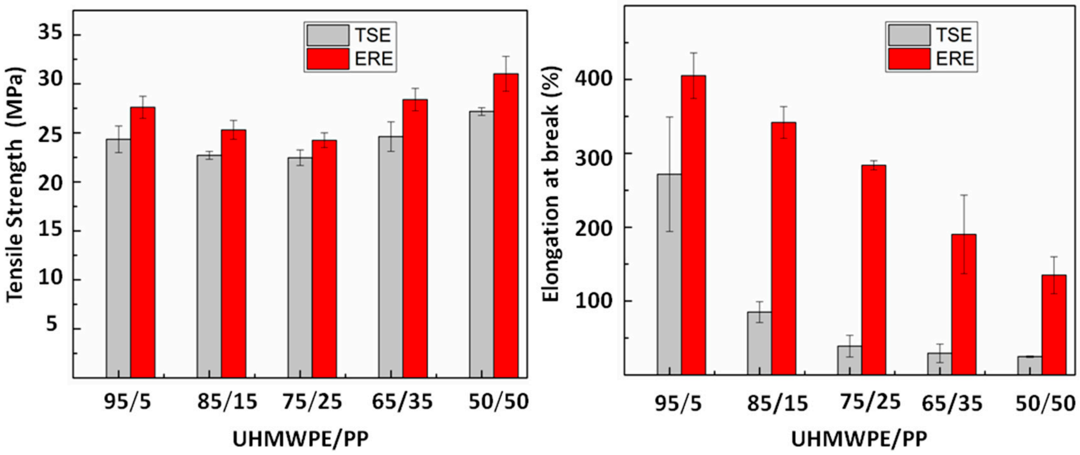

Figure S4. Mechanical properties of UHMWPE/PP under different processing methods. (a) tensile strength; (b) elongation at break.

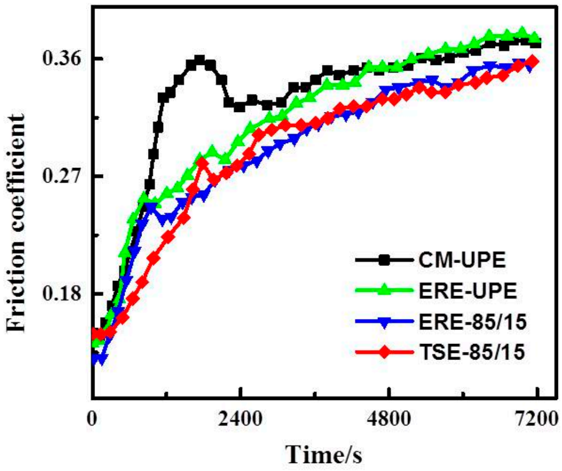

Figure S5. Curve of friction coefficient as a function of friction time of UHMWPE and UHMWPE/PP under different processing methods.

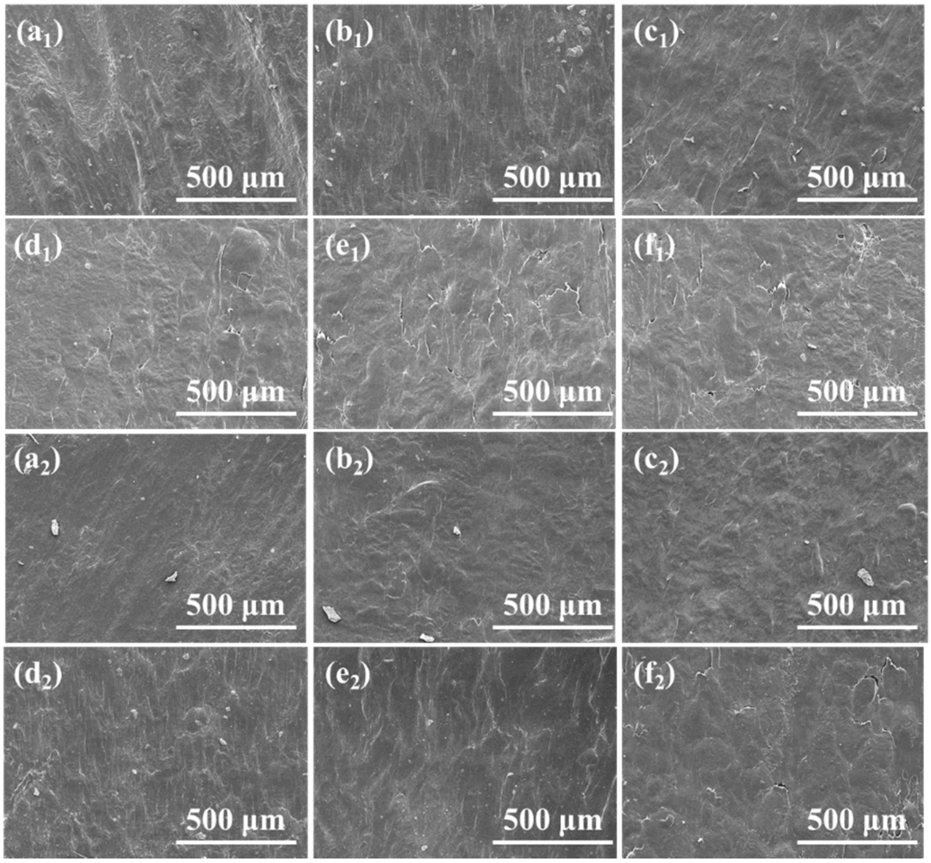

Figure S6. SEM images of the end of the friction test of UHMWPE and UHMWPE/PP: (a) 100/0; (b) 95/5; (c) 85/15; (d) 75/25; (e) 65/35; (f) 50/50. "1" and "2" stand for the UHMWPE/PP prepared by shear flow and elongational flow respectively ; a_1 stands for pure UHMWPE prepared by direct molding.

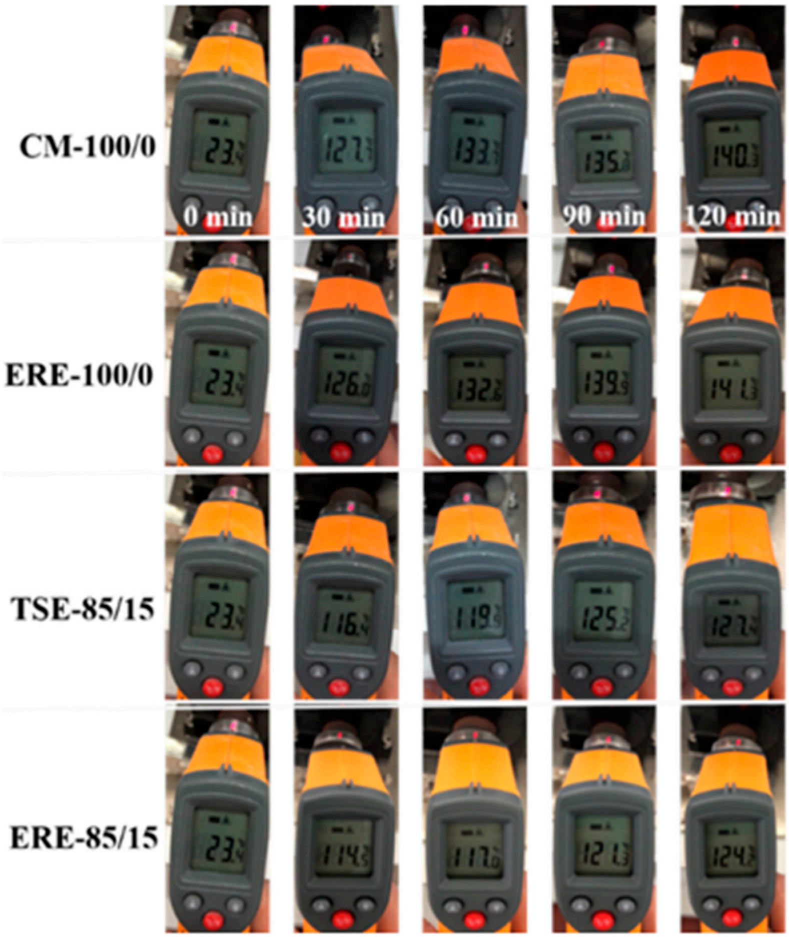

Figure S7. Friction temperature of UHMWPE and UHMWPE/PP under different processing.

Table S1. DSC related data of UHMWPE by different processing methods.

**ERE**	**T_c（℃）**	**T_m（℃）**	**ΔH_m（J/g）**	**X_c（%）**
100/0	116.7	135.3	151.6	52.3
95/5	115.2	134.6	141.2	51.2
85/15	117.7	134.6	122.8	49.8
75/25	117.1	134.9	107.3	49.3
65/35	114.2	135.8	82.8	43.9
50/50	114.6	134.8	60.9	42.0
**TSE**	**T_c（℃）**	**T_m（℃）**	**ΔH_m（J/g）**	**X_c（%）**
100/0	116.4	134.8	149.9	51.7
95/5	117.4	134.1	136.2	49.4
85/15	117.8	133.9	121.3	49.2
75/25	117.9	133.7	96.9	44.5
65/35	117.9	133.8	82.1	43.5
50/50	118.2	133.4	59.4	41.0

Table S2. Surface frictional temperature by different friction time (°C).

Sample	0 min	30 min	60 min	90 min	120 min
CM-100/0	23.4	127.7	133.7	135.8	140.3
ERE-100/0	23.4	126.0	132.6	139.9	141.3
TSE-85/15	23.4	116.4	119.9	125.2	127.4
ERE-85/15	23.4	114.5	117.0	121.3	124.2
